# ENVEJECIMIENTO Y ESTRATOS: SOCIOECONOMIC INEQUALITY IN AGING IN COLOMBIA

**DOI:** 10.1093/geroni/igz038.2901

**Published:** 2019-11-08

**Authors:** Margarita Osuna, Jennifer A Ailshire

**Affiliations:** University of Southern California, Los Angeles, California, United States

## Abstract

Colombia has the highest level of income inequality among Latin American countries, which likely translates into disparities in the aging experience. This study uses data on adults ages 60 and older from the 2017 National Survey of Quality of Life to examine socioeconomic stratification in physical health and psychological well-being. Colombians are assigned to estratos that reflect their residential location as well as social and economic position. Compared to those in the lowest estrato, older adults in the middle and high estratos are less likely to report having sensory impairment or difficulty with daily activities. They are also 1-2 times more likely to report feeling happy and calm. Those in the highest estratos are less likely to report feeling worried. Results suggest there is tremendous variation in the aging experience across socioeconomic strata and that older adults in the lowest strata are particularly disadvantaged with respect to health and well-being.

